# Robust Image Reconstruction Strategy for Multiscalar Holotomography

**DOI:** 10.3390/jimaging8020037

**Published:** 2022-02-02

**Authors:** Maximilian Ullherr, Matthias Diez, Simon Zabler

**Affiliations:** Lehrstuhl für Röntgenmikroskopie, Universität Würzburg, Josef-Martin-Weg 63, 97074 Würzburg, Germany; maximilian.ullherr@posteo.de (M.U.); matthias.diez@uni-wuerzburg.de (M.D.)

**Keywords:** reconstruction, region of interest, ROI, multiscalar holotomography, holotomography, computed tomography, CT, X-ray

## Abstract

Holotomography is an extension of computed tomography where samples with low X-ray absorption can be investigated with higher contrast. In order to achieve this, the imaging system must yield an optical resolution of a few micrometers or less, which reduces the measurement area (field of view = FOV) to a few mm at most. If the sample size, however, exceeds the field of view (called local tomography or region of interest = ROI CT), filter problems arise during the CT reconstruction and phase retrieval in holotomography. In this paper, we will first investigate the practical impact of these filter problems and discuss approximate solutions. Secondly, we will investigate the effectiveness of a technique we call “multiscalar holotomography”, where, in addition to the ROI CT, a lower resolution non-ROI CT measurement is recorded. This is used to avoid the filter problems while simultaneously reconstructing a larger part of the sample, albeit with a lower resolution in the additional area.

## 1. Introduction

Holotomography [1,2] is a 3D imaging technique for laboratories and synchrotrons that produces high contrast images of (a) low-density samples or (b) multi-material objects with at least one low-contrast material interface. Unlike attenuation contrast computed tomography, which does not allow for the analysis of these materials, holotomography allows for the imaging of those kinds of samples with high contrast. Recording holotomography requires one or several high-resolution (1–5 μm or less) scans with a finite propagation distance *d*, which results in Fresnel fringes highlighting both the high- and the low-contrast material interfaces. Those so-called (in-line) phase contrast images are converted by a so-called phase retrieval algorithm into an absorption-like image with a higher contrast. Before or after this step, the 3D volume image is built from its projections, e.g., by a standard filtered back projection (FBP).

As a consequence of the high resolution requirement, the effective field of view (FOV) of the measurement is restricted to some mm at most. For certain materials (e.g., biological soft tissue, plants, or composite material, such as carbon fiber reinforce plastics (CFRP)), cutting or grinding the sample down to this size is not desirable, mostly because such a preparation would destroy or at least severely alter the structure. Alternatively, one could record a region of interest computed tomography (ROI-CT), which is an imaging mode where the sample size exceeds the FOV in the horizontal detector dimension.

ROI-CT poses an identical problem for both of the Fourier filters in the phase retrieval and in the filter part of the filtered backprojection (FBP) algorithm of the computed tomography reconstruction. To understand this better, consider that Fourier filters can also be viewed as real space convolutions, where the real space convolution kernel leads to the concept of filter range. Filter range is the distance from a point in the filtered image, where the values of the original image strongly influence the filter result at that point. This range is different for different Fourier filters. At the edge of an image, this means that an area with the width of the filter range (artifact band) cannot be filtered correctly, because the correct values beyond the image edge are not known (measured). Effectively, the usable measurement area becomes smaller by the area of the artifact band.

To avoid this border value problem, one tries to estimate the values beyond the image edge and use this estimate in the filter. Depending on how good this estimate is, the actual width of the artifact band may be much smaller than the filter range. The aim is then to reduce the artifact band by so much that it is practically irrelevant. In the context of computed tomography, the artifact band lies at the edge of the projections or at the outer surfaces of the cylindrical reconstructed volume image (rim and top/bottom).

Another approach is multiscalar holotomography, where we measure the values beyond the image edge, albeit with a lower spatial resolution. To carry this out, we record two holotomography scans, one with high resolution (HR) and a small FOV (=ROI-scan), and the other with a larger FOV but lower resolution (LR). Both scans are combined in order to build an artifact-free volume image that combines the high resolution in a part of the image with a large FOV. The main challenge lies in merging the two data sets without creating additional artifacts. In addition to avoiding ROI artifacts in the HR area, the resolution can also be improved in the LR area in the vicinity of the ROI area. The two scans can, in principle, be recorded simultaneously, but for this study, were recorded after each other.

## 2. Theory and Methods

We will reconstruct a multiscalar holotomography in the following steps: (1) Obtain the two holotomography data sets with different spatial resolutions/FOVs but the same number of projections. (2) Determine the relative scale and the shifts between the data sets. (3) Interpolate the lower resolution projections to the higher resolution and merge the projections to multiscalar projections. (4) Reconstruct a volume image. (5) Apply phase retrieval. Alternatively, phase retrieval may be carried out after step (3).

Changing the spatial resolution is easy for experimental setups, both at a synchrotron beamline and the laboratory. In the laboratory, one can record scans at different optical magnifications, while a synchrotron imaging beamline usually has a set of detector configurations to allow for different resolution/FOV settings.

In the multiscalar holotomography, the computed tomography and phase retrieval filters do not suffer from ROI artifacts if the lower resolution scan is not a ROI scan. If the LR scan is a ROI scan, the ROI artifacts are at least moved outside of the HR area.

Alternatively, one could only extend the HR projection with the data from the LR data set to avoid ROI artifacts, reconstruct both data sets separately, and merge the volume reconstructions. This method might be even simpler and better suited for large differences in the lower and higher resolution.

### 2.1. Data Set Merge

For a correct interpolation of the LR data set (step 3), the exact relative scale factor and the relative shift must be known. The relative scale factor can, e.g., be determined by generating multiscalar holotomographies with different scale factors and selecting the one where scale artifacts are minimal. It would be best to also determine the relative scale experimentally to a high precision, e.g., with metric phantoms, which was not carried out here.

The relative shift can be determined by correlating two images from the two data sets. Here, we calculate the standard deviation of the difference in two images $I_{HR}$ and $I_{LR}$, where Δx and Δy are the relative shifts and $I_{LR}\left(\Delta x,\Delta y\right)$ is the interpolated and shifted projection. The correct shifts are assumed when the standard deviation is minimal:

(1) $${\left(\Delta x,\Delta y\right)}_{\mathrm{correct}}=\mathrm{argmin}\left\{\mathrm{std}[I_{HR}-I_{LR}\left(\Delta x,\Delta y\right)]\right\}$$

The correct shift is then determined for a number of projections and the results are averaged.

Note that, if the rotational axes of the two data sets were chosen differently, the horizontal shift Δx follows a projected circular motion, which can be written as:

(2) $$\Delta x=a\sin\left(\phi -\phi_{0}\right)+x_{0},$$

where ϕ is the rotational angle of the sample, *a* the shift amplitude, $\phi_{0}$ the angle offset, and $x_{0}$ the shift offset. In this case, the relative shifts are determined for a set of angles between 0${}^{\circ }$ and 180${}^{\circ }$/360${}^{\circ }$. Equation (2) is fitted to these values. In practice, even the data sets that were experimentally set up to have the same rotational axis may show small differences in the actual rotational axis (in this study, *a* = 1–3 pixels).

### 2.2. Merge Artifact Removal

By applying the phase retrieval after the merge (step 3), both ROI-CT and ROI phase retrieval artifacts can be avoided. Nevertheless, there may appear new merge artifacts because the gray values of the LR and HR data sets at the ROI edge may not match closely enough. Due to the fact that we apply phase retrieval, any small-scale artifacts are heavily suppressed, and are effectively treated as phase contrast features and filtered out. This leaves the large-scale artifacts to be corrected. These usually stem from small errors in the flat field correction, e.g., if the X-ray source intensity fluctuates.

Here, we remove the large-scale artifacts by inserting part of the large-scale information of the lower resolution (LR) data set into the higher resolution (HR) data set before the merge. The strength of the correction is given by the relative adaption strength *b*. The large-scale information of the LR field projection $P_{\mathrm{LR}}$ is calculated by filtering in Fourier space with a low pass filter l(b). The corresponding large-scale information in the HR projection $P_{\mathrm{HR}}$ is removed by a corresponding high pass filter h(b)=1−l(b). Thus, if both data sets were images of the same object and we sum both filter results, we again obtain a valid image of the object, the corrected HR image ${\tilde{P}}_{\mathrm{HR}}$:

(3) $${\tilde{P}}_{\mathrm{HR}}=F_{2D}^{-1}\left\{h\left(b\right)F_{2D}\left\{P_{\mathrm{HR}}\right\}\right\}+F_{2D}^{-1}\left\{l\left(b\right)F_{2D}\left\{P_{\mathrm{LR}}\right\}\right\}$$

For low pass and high pass filters, we used smoothed Heaviside step functions H(u). The smoothing is necessary to avoid ripple artifacts and is carried out by convolution (∗) with a Gaussian filter kernel *g*. We define:

(4) $$h\left(b\right)=H\left(u-u_{b}\right)*g;\;l\left(b\right)=H\left(u_{b}-u\right)*g,$$

where $u_{b}$ is the cutoff frequency of the low pass/high pass. The condition h(b)=1−l(b) is satisfied because:

(5) $$H\left(u-u_{b}\right)+H\left(u_{b}-u\right)=1\;\Rightarrow \;h\left(b\right)+l\left(b\right)=1*g=1,$$

using the exchangeability of the sum and the convolution operation.

We define the relative adaption strength *b*:

(6) $$b=\frac{u_{b}}{u_{ny}};\;b\in \left[0,1\right]$$

where $u_{ny}$ is the Nyquist frequency of the LR data set.

### 2.3. Phase Retrieval

We use the phase retrieval algorithm described in [3,4], but instead of applying the main step (the Fourier filter) on projections, we apply the filter to the volume image. For multi-material samples, the extension described in [5] is used. The phase retrieval Fourier filter kernel is:

(7) $$K_{p}\left(u\right)=\frac{1}{1+p^{2}u^{2}}$$

where $u^{2}=u_{x}^{2}+u_{y}^{2}+u_{z}^{2}$ is the radial spatial frequency in three dimensions. The strength of the phase retrieval is given by *p*, which has the unit of a length and whose value gives an estimate of the filter range. The value for *p* can be calculated from material constants, but here, we determine it empirically. To do so, filters with different values of *p* are applied to volume images and the volume image with the best visual result is selected to determine *p*. Applying the phase retrieval after the FBP has the additional advantage of suppressing high frequency image artifacts from the FBP algorithm. These are worse for the multiscalar computed tomography than usual because interpolating the LR data set to the higher resolution leads to a reconstruction with fewer projections and a larger angle step than optimal.

If one of two data sets needs a different value for *p*, a kernel of the following form must additionally be applied to only its projections:

(8) $$K_{p\to \tilde{p}}\left(u\right)=\frac{1+p^{2}u^{2}}{1+{\tilde{p}}^{2}u^{2}}$$

which effectively only applies $K_{\tilde{p}}$ to this data set, because $K_{p}$ is inverted by $K_{p\to \tilde{p}}$.

### 2.4. Padding

Padding is a process where, before applying a Fourier filter to an image, the image is extended at the image edges. Padding is routinely applied when using a FFT algorithm to avoid the opposite edges of the (actual) image influencing each other. For ROI phase retrieval or ROI-CT, the padding tries to approximate the structure outside of the image in order to to minimize image artifacts at the edge (artifact band). Different padding methods use different algorithms for this purpose, but all are approximations and there is no “correct” way to carry this out.

The padding methods we tested are: (a) Zero padding—filling the outer area with zeros. (b) Edge padding—filling the outer area column-/row-wise with the values at the edge pixels (e.g., used in ANKAphase [6]). (c) Reflect padding—filling the image area outside with the same values as inside, but reflected at the edge.

We will call method (d) normalize padding. It is carried out in the following steps: (1) Filter the image with zero padding. (2) Filter an image with all pixels set to unity with zero padding. (3) Divide the first by the second to obtain the result. The second image essentially gives the weighted fraction of pixels affected by the filter actually inside the image. By dividing by it, we restore the correct normalization to the filter if the zero padded (“outside”) pixels were not counted. This method can easily be applied even when the “inside” and “outside” areas have arbitrary geometries. Here, we use it in the case of ROI volume reconstructions, where the “inside” is the cylindrical shape inside a rectangular volume. We also use it in an extension of the multi-material phase retrieval [5], where “inside” is the area where the filter is applied.

We note that zero padding is commonly used, but it is a very bad approximation for phase retrieval (and also in most other cases, e.g., the filter step in a ROI-FBP). As a result of the usually low X-ray absorption in phase contrast measurements, the gray values at the image edge are close to unity, which is not well approximated by zero. This results in very strong filter artifacts.

### 2.5. Filter Ranges

In the following, we analyze the real space filters that are part of the phase retrieval and FBP algorithms. The important quantities are the filter ranges, because they give the width of the artifact band.

The real space convolution filter of the phase retrieval filter Equation (7) is:

(9) $$k_{p}\left(\Delta x\right)\propto \exp\left(-2\pi \Delta x/p\right)$$

This represents a low-pass filter and has a range of ≈*p* (Δx is the distance between the result and image pixels).

The real space convolution filter of the ramp filter of the filtered backprojection [7] is:

(10) $$k_{r}\left(\Delta x\right)=\left\{\begin{array}{cc} 1/4 & \mathrm{for}\;\Delta x=0\\ -{\left(\pi \Delta x\right)}^{-2} & \mathrm{for}\;\Delta x\;\mathrm{uneven}\\ 0 & \mathrm{else} \end{array}\right.$$

where Δx is an integer distance. This filter is a combination of a high pass and a long-ranged low pass (uneven indices). The low pass part is the one that generates an artifact band. Through experimentation with our own implementation of the FBP and estimates from Equation (10), we found that the filter has a range of roughly 200 pixels, but strongly visible artifacts are limited to a range of <50 pixels. The following backprojection part does not generate an additional artifact band.

## 3. Application

### 3.1. Methods

The measurements were recorded at the ID19 beamline at the European Synchrotron Radiation Facility (ESRF) in Grenoble, France. The LR scans were recorded with an effective pixel size of 2.7 μm, and the HR scans with a effective pixel size of 1.1 μm. All data sets were recorded with a polychromatic wiggler spectrum with an average photon energy of 19 keV and 1500 projections with a detector size of 2016 × 2016 pixels for HR and 1536 × 2016 pixels for LR. In addition to the phase retrieval, a Wiener Gaussian deconvolution was applied to improve image sharpness, assuming a standard deviation of 1.2 μm for both scans. The deconvolution was not used for the phase contrast images, as they are already oversharpened. The computed tomographies were reconstructed with the software Octopus (version 8.7) from the company Inside Matters (Belgium). For the ROI-CT reconstructions, the ROI-CT filter of this software was used, which also estimates padding based on the data. The ring filter was used for the non-ROI data sets (it cannot be used for ROI data sets).

The two samples:

- The rose stem sample is a dry rose stem with a diameter of approximately 3 mm. The interesting interface with the low absorption difference is between air and wood. The sample also contains small calcium oxalate [8] (CaOx) crystals. For this sample, the multi-material phase retrieval method from [5] is used to phase retrieve both the air–wood (*p* = 60 μm) and the air–CaOx (*p* = 20 μm) interfaces correctly.
- The CFRP sample consists of a small plate of carbon-fiber-reinforced plastic with a thickness of approximately 0.25 mm and a fiber diameter of approximately 6 μm. For phase retrieval, *p* = 70 μm was used. Here, even the large FOV scan does not fully cover the sample length, and hence remains an ROI scan.

### 3.2. Simple Reconstructions

**Figure 1:**
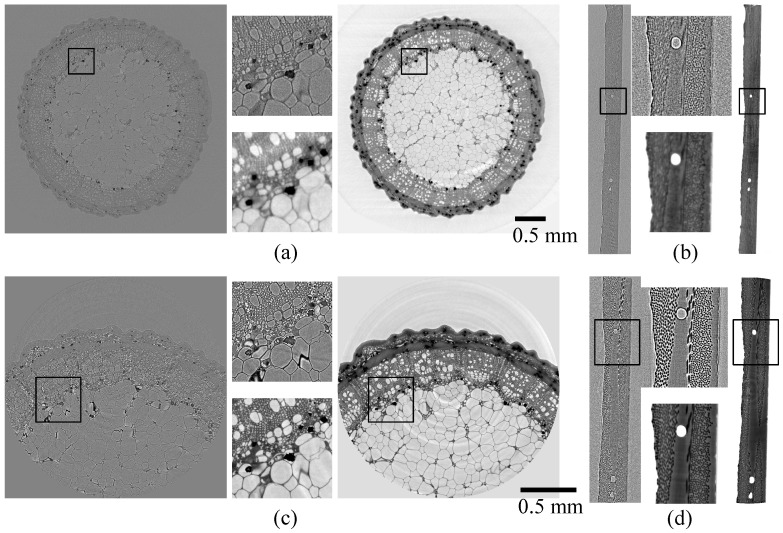
Volume slices with and without phase retrieval with enlarged insets. Images without phase retrieval (phase contrast tomography, gray background) are left/above and with phase retrieval (holotomography, white background) are right/below. Rose stem sample: (**a**) LR, (**c**) HR; CFRP sample: (**b**) LR, (**d**) HR. As is usual for holotomography, ring artifacts are the most prominent artifacts present. The scale is identical for all ROI and for all large FOV scans; the insets are identical areas for ROI and large FOV and have a side length of 0.4 mm.

Figure 1 shows volume slices for the LR and HR scans (no merge) of the two samples with and without phase retrieval. The LR projections were interpolated to the resolution of the HR projections. The visual interpretation is much easier for the phase retrieved images, and they can also easily be segmented, whereas the images without phase retrieval cannot.

The ROI holotomographies (b–d) show neither ROI-CT nor ROI phase retrieval artifacts in a problematic way. The artifact band at the rim of the images is less than 5 pixels wide. Note that projection-based phase retrieval with a good padding method would work even better, but the (multi-material) phase retrieval method used needs to be applied as a volume filter after the FBP reconstruction.

### 3.3. Test of the Merge Artifact Removal

**Figure 2:**
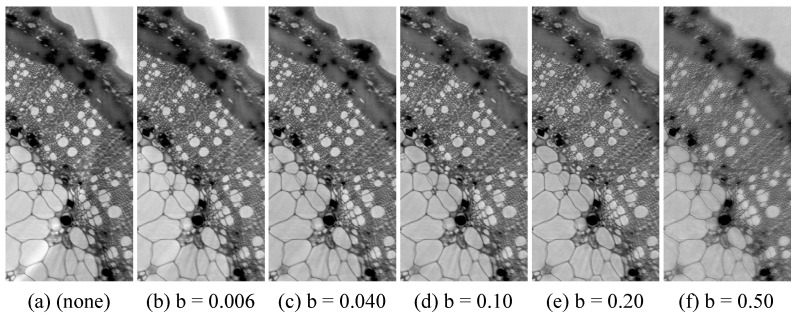
Volume slices of multiscalar holotmographies at the left edge of the ROI area for testing the merge artifact removal. Without adaption (**a**) and for increasing adaption strengths *b* (**b**–**f**).

Figure 2 shows multiscalar holotomographies for different strengths of the merge artifact removal method, characterized by the adaption strength *b*. The area shown is at the left edge of the ROI area and the merge artifact appears only at the edge of the ROI and is similar for the whole edge. For comparison, in Figure 2a, the merge artifact removal was not applied. Figure 2b–f show an increasing adaption strength. Image (b) corresponds to an adaption value too low for an effective artifact removal, whereas, in (e–f), the value is too high and produces small-scale artifacts. From these images, *b* = 0.1 was selected for the following multiscalar holotomographies.

### 3.4. Multiscalar Holotomography

**Figure 3:**
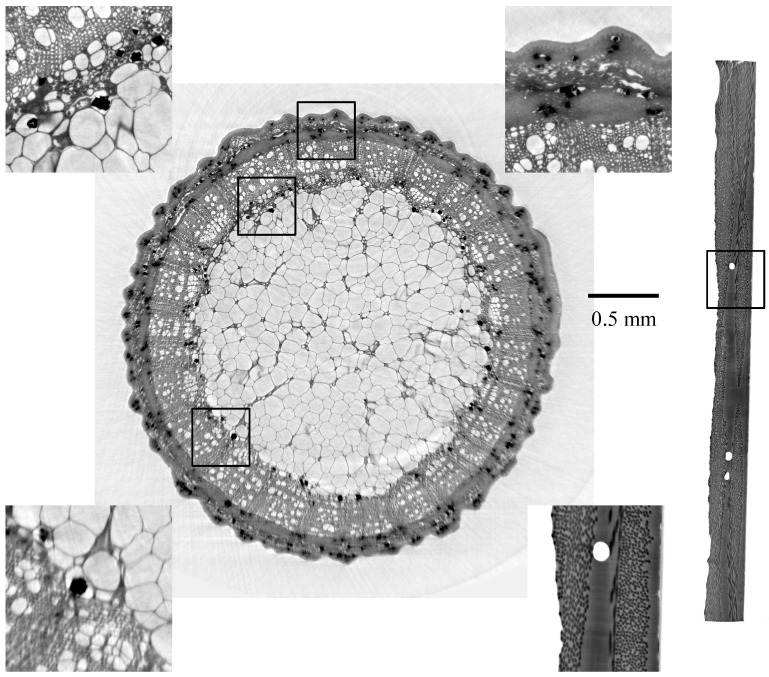
Volume slices of multiscalar holotmographies, rose stem sample (left, ROI area to the top), CFRP sample (rigth, ROI area centered). Compare to Figure 1 for the exact ROI areas. The upper two insets are from the ROI area of the rose stem sample, the lower left inset is outside the ROI area. The upper left and the lower right inset are from the same areas as in Figure 1. Scale bars are identical for both samples.

The multiscalar holotomographies of the two samples are shown in Figure 3, with phase retrieval applied to the volume images. They show images where the original HR/ROI area is reconstructed with the higher spatial resolution, and yet the FOV of the image is that of the LR scan. Note that the higher resolution of the ROI improves the result beyond the original ROI area, giving an improved resolution in a much larger area.

### 3.5. Comparison of Padding Methods for ROI Phase Retrieval

Finally, we now want to investigate how well different padding methods mitigate ROI phase retrieval artifacts. To achieve this, we want to avoid other similar artifacts: ROI-CT and merge artifacts.

This is achieved by reconstructing multiscalar holotomographies, but with the phase retrieval applied prior to the merge. For the phase retrieval step of the HR data set, different padding methods (see Section 2.4) are then tested. The phase retrieval filter acts on the HR/ROI and the LR data sets separately, but the merge artifact removal is used and the FBP reconstruction is applied to the merged projections. As a consequence, the results shows ROI phase retrieval artifacts, but neither ROI-CT nor merge artifacts. The disadvantage of this approach is that the ROI phase retrieval artifacts are distorted (slightly) by the merge artifact removal (*b* = 0.05).

**Figure 4:**
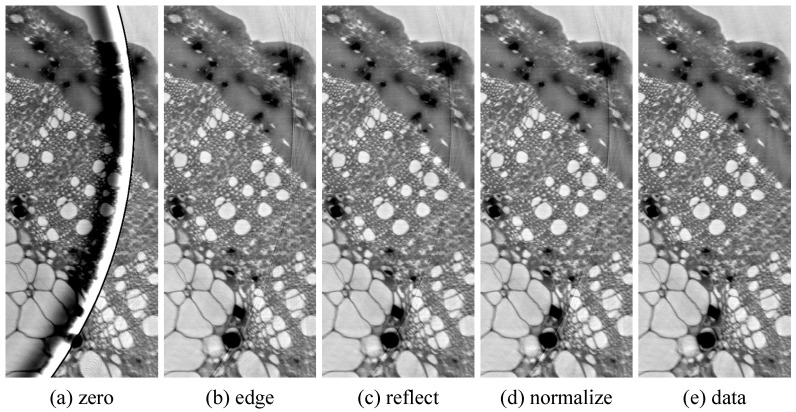
Volume slices of multiscalar holotmographies at the left edge of the ROI area with different padding methods for phase retrieval at the ROI rim (**a**–**d**) and, for comparison, without ROI phase retrieval artifacts (**e**). The artifact in (**a**) is distorted by the merge artifact removal; the FBP filter would produce a similar artifact band with longer range but lower intensity.

Figure 4 shows volume slices with different padding methods applied. In (a), we see that zero padding is as bad an approximation as expected. These artifacts are also heavily distorted by the merge artifact removal. We note that padding with a constant value of 0.8 (≈projection mean) would also mostly avoid artifacts here. Figure 4b–d show artifacts only in the immediate vicinity of the ROI rim (few pixels), with the edge padding slightly worse than the other two. Figure 4e was produced by applying phase retrieval after the merge (padding with the actual data); it is there for comparison because it does not have ROI phase retrieval artifacts. Note that the streak artifacts originating from the image edge are outside of the ROI area and would not appear in a ROI holotomography of the HR data set.

## 4. Conclusions

In a situation where holotomography must be a ROI-CT, we conclude from our examples that the possible problem of a large artifact band in the reconstructed volume image is avoidable through good padding for the filter steps in the phase retrieval and the FBP. Due to the fact that several different and simple padding methods work well, we conclude that good padding is not difficult if the associated problems are considered carefully.

We demonstrated that multiscalar holotomography is a more thorough approach to avoid ROI artifacts and is a good solution when both a high spatial resolution and a large field of view are desired. Its disadvantages are the higher degree of complexity in the reconstruction and the need to record two measurements for one sample.

## Data Availability

Not applicable.

## Abbreviations

The following abbreviations are used in this manuscript:

FOV	field of view
ROI	region of interest
CT	computed tomography
FBP	filtered back projection
CFRP	carbon-fiber-reinforced plastics
ROI-CT	region of interest computed tomography
HR	high resolution
LR	lower resolution
CaOx	calcium oxalate crystals
